# A New Azimuth-Dependent Elevation Weight (ADEW) Model for Real-Time Deformation Monitoring in Complex Environment by Multi-GNSS

**DOI:** 10.3390/s18082473

**Published:** 2018-07-31

**Authors:** Junqiang Han, Guanwen Huang, Qin Zhang, Rui Tu, Yuan Du, Xiaolei Wang

**Affiliations:** 1 College of Geology Engineering and Geomantic, Chang’an University, 126 Yanta Road, Xi’an 710054, China; 2015026002@chd.edu.cn (J.H.); 2017026010@chd.edu.cn (Y.D.); 2015026004@chd.edu.cn (X.W.); 2 National Time Service Center, Chinese Academy of Sciences, Shu Yuan Road, Xi’an 710600, China; turui@ntsc.ac.cn

**Keywords:** GNSS, ADEM, ADEW, monitoring, azimuth, elevation mask

## Abstract

Global navigation satellite systems (GNSS) have provided an excellent way to monitor micro-deformation in real-time. However, at local sites where landslides frequently occur, the environment can include complex surroundings with mountains, dense vegetation, and human settlements, which can severely degrade the accuracy of positioning with the GNSS technique. In this study, we propose an azimuth-dependent elevation weight (ADEW) model using an azimuth-dependent elevation mask (ADEM) to reduce the effects of multipath errors and improve the accuracy of real-time deformation monitoring in such environments. We developed an adaptive fixed-elevation mask to serve as the outlier of low precision observations at lower elevations for the ADEM, and then, we applied the weighted phase observations into the mitigation process for the effects of multipath errors. The real numerical results indicate that the ADEM model performs better than the conventional model, and the average improvements were 18.91% and 34.93% in the horizontal and vertical direction, respectively. The ADEW model further improved upon the ADEM model results by an additional 21.9% and 29.8% in the horizontal and vertical direction, respectively. Therefore, we propose that the ADEW model can significantly mitigate the effects of multipath errors and improve the accuracy of micro-deformation monitoring via GNSS receivers.

## 1. Introduction

Many countries around the world suffer from recurring geological disasters, especially landslides, and the losses of lives and properties caused by the latter have increased each year. Presently, there is an urgent need for the development of high precision, real-time landslide monitoring technology. Global Navigation Satellite Systems (GNSS), which capture global, continuous, and high precision geospatial data, have been widely applied in many fields [1,2,3,4,5,6,7], especially for the monitoring of geological hazards [8,9,10,11,12]. Given the excellent efficiency and reliability, real-time kinematic (RTK) techniques based on short-baselines have been implemented in geologic deformation monitoring over the past few years [13,14]. However, local obstacles surrounding antennas can lead to both diffraction and multipath errors. These have been recognized as the major sources of errors that impact the accuracy of positioning [15].

Various studies have explored techniques for mitigating the effects of diffraction and multipath errors, and these techniques can be classified into three groups. The first group usually mitigates multipath errors based on antennas [16,17], for example by using choke antennas, but only a portion of the multipath effects can be eliminated by this technique. The second group of techniques is based on stochastic variance models [18,19,20]. The SIGMA-ε variance model was developed by Hartinger et al. [18] with the aim of mitigating the diffraction error effects with high precision. In 1999, Hartinger et al. [19] developed the stochastic SIGMA-Δ model to eliminate the effects of diffraction errors. However, these models appear to fail when there is an unknown number of reflecting objects and when there are interactions among diffraction pathways. To overcome these limits, Wieser and Brunner [21] proposed an extended weight model for global positioning system (GPS) phase observations, but additional iterations are needed to detect robust errors. The third group depends on modeling techniques that use the signal-to-noise ratio (SNR) [21,22,23,24,25] or on filtering techniques [26,27], such as the Kalman filter [28], wavelet analysis [29], Vondrak filter [30,31], and Sidereal Filter (SF) [32,33,34,35,36,37,38,39,40]. Regarding the SNR-based methods, they have proven to be effective only when dealing with multipath errors caused by single objects, and the approaches based on filtering techniques have been proven effective for post-processing applications.

Presently, the typical SF model and Multipath Hemispherical Map (MHM) model are used routinely for deformation monitoring. Dong et al. [39] proposed mitigating multipath errors with single-difference observations using the MHM model, but a common receiver clock is needed, as well as precise atmospheric products. Others have suggested the use of the MHM model with double-difference observations from the BeiDou Navigation Satellite System (BDS) for multipath error mitigation [40,41]. However, both methods have an effective reduction capacity for multipath errors that relies on the similar spatial repeatability of orbit traces within few days.

Few studies involving the construction of physical-based models for GNSS have been validated in complex environments, which are typical environments where landslides occur. Klostius et al. [42] suggested that an azimuth-dependent elevation mask (ADEM) model could be used in conjunction with conventional theodolite measurements. Atilaw et al. [43] proposed the development of an azimuth-dependent elevation threshold (ADET) mask via GNSS data to reduce the multipath errors in ionospheric studies, noting that an optimum physical elevation mask could be modeled with high precision by GNSS satellites. Landslides frequently occur in complex environments, and these sites are typically surrounded by vegetation, human infrastructures and mountain ridges. As a result, it is not convenient to map the horizontal mask with theodolite measurements. Without considering the deviations of source error caused by different types of vegetation and topography, we propose the use of an azimuth-dependent elevation weight (ADEW) method based on an ADEM in a stochastic model for mitigating the effects of both diffraction and multipath errors. With such a method, it may be possible to achieve positional accuracies at the millimeter-scale in complex environments.

This manuscript is organized as follows. First, the models for the double-difference technique, which adopts RTK methods, the ADEM, and the ADEW, are introduced. Then, the performance of the ADEW model is evaluated and discussed after experimentation in real landslide regions. Finally, we provide our conclusions and suggestions for future works.

## 2. Models

The double differencing (DD) technique allows for the elimination of most orbital, tropospheric delay, and ionospheric delay errors in short-baseline relative positioning. Consequently, this technique has been widely used for short-baseline positioning.

### 2.1. DD Measurement Model for Short-Baselines

Inter-system biases among different navigation systems can be neglected for short-baseline positioning [44]. Because of this, the DD carrier-phase model for a single satellite can be expressed as follows [45]:

(1) V=∇Δφ−(∇Δρ+∇ΔM+∇ΔN+∇Δε),

where ∇Δ is the DD operator, and ∇ΔM and ∇ΔN are the DD multipath effects and the integer ambiguities, respectively. ∇Δφ denotes the DD observed phase, and ∇Δρ denotes the DD distance between satellites and receivers. Finally, ∇Δε and V are the noise and DD residuals (Res), respectively. The unit of all terms is meters (m).

The elevation stochastic model used in this study for static baseline solutions is expressed as $\sigma^{2}=a^{2}+b^{2}/\sigma_{e}^{2}$, where $\sigma_{e}^{2}=\sin^{2}\left(E\right),$ and E is the elevation angle of the satellite. a and b denote the carrier-phase error factor, and the default value was set as 0.003 m [40]. The variance of the observations is denoted as $\sigma^{2}$. With the float values of integer ambiguity calculated by a least-squares estimator, the integrated ambiguity can be determined by the possibility of Lambda [45,46,47]. The fixed solution is then calculated by the fixed ambiguity.

### 2.2. ADEM Model

**Figure 1:**
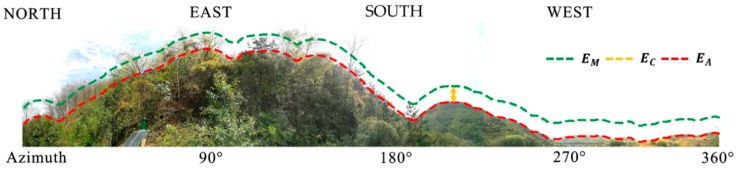
Panoramic view of the study environment surrounding the antenna from 0°–360° along the azimuth in the landslide region, as well as the meanings of the terms $E_{M}$ (green), $E_{C}$ (yellow), and $E_{A}$ (red).

It has been previously demonstrated that the signals of GNSS can be potentially obstructed by concrete obstacles (i.e., topographic ridges) as well as by disperse obstacles (i.e., trees), as is shown in Figure 1. Note that the maximum bias on the carrier-phase was due to the diffraction of ~7 cm [42] and that the bias due to the multipath on frequency L1 was ~4.8 cm [48]. Traditionally, a typical fixed cut-off elevation (TFC) (15°–30°) dependent on the geoid is employed to reduce the multipath errors, as it is assumed that the antenna is mounted in an unsheltered environment. However, an antenna may be mounted in a complex environment as shown in Figure 1.

Where $E_{M}$ denotes the changeable cut-off angle dependent on the azimuth, $E_{C}$ denotes the constant cut-off elevation, and $E_{A}$ denotes the specific elevation mask.

Hence, the ADEM model is proposed, whereby instead of the geoid approach, the model uses a specific azimuth-dependent elevation mask. The ADEM model can be expressed as follows:

(2) $$E_{M}=E_{C}+E_{A},$$

**Figure 2:**
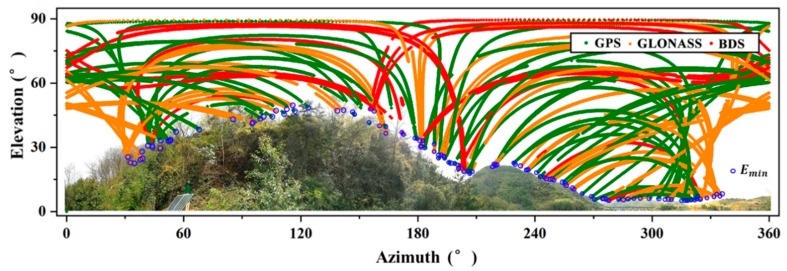
All the trajectories of GNSS satellites in the elevation and azimuth domain of 24 h corresponding to Figure 1, as well as the scatter pointers $E_{min}$.

In Equation (2), $E_{M}$ is decided by $E_{A}$, when $E_{C}$ is set as a constant value. The main challenge of the ADEM model is to derive $E_{A}$ in a convenient way. Note that when the satellite rises and sets behind a mountain ridge, the line-of-sight between the satellites and receiver can be interrupted. As a result, the elevations of the initial and terminal points ($E_{min}$) in the trajectory approximately intersect with the mountain ridge. Therefore, the scatter of $E_{min}$ for GNSS can be employed to describe the physical horizon $E_{A}$ surrounding the antenna. Here, data were collected from GNSS receivers mounted in the study environment during a 24 h session. All the trajectories of GNSS satellites in the elevation and azimuth domain are shown in Figure 2.

It can be noted from Figure 2 that the physical structures surrounding the antennas can be described precisely by the $E_{min}$ of the multi-GNSS, although a slight discrepancy with the photograph may be observed. Based on the $E_{min}$ of 24 h, the curve fitting package in MATLAB was adopted to estimate the fitting coefficients for $E_{A}$. Then, the $E_{A}$ can be expressed as follows:

(3) $$\{\begin{array}{l} \vec{P}=polyfit\left(\vec{x},\vec{y},n\right)\\ E_{A}=polyval\left(\vec{P},x_{i}\right) \end{array},$$

where $\vec{x}$ and $\vec{y}$ respectively denote the azimuth and elevation angle vector of $E_{min}$ in 24 h, and $\vec{P}$ represents the coefficients for the polynomial of degree n. The functions polyfit and polyval are in the curve fitting package. The $\vec{P}$ coefficients can be derived by polyfit, while the $E_{A}$ on azimuth $x_{i}$ of any line-of-sight can be recalculated from $\vec{P}$ by polyval.

### 2.3. ADEW Model

**Figure 3:**
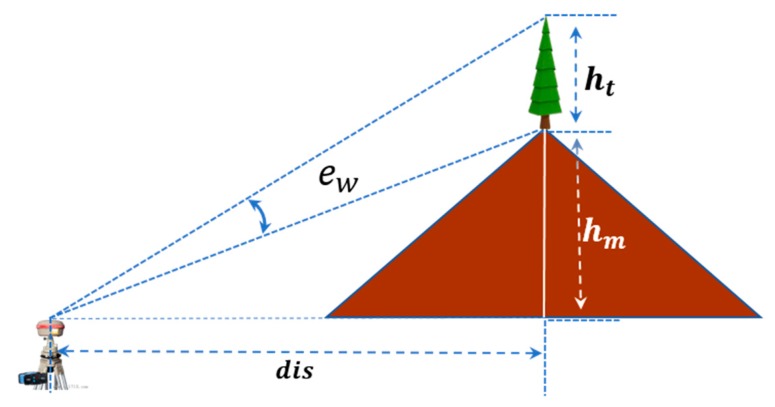
The geometric relationships among the specified range ($e_{w}$), height of a mountain peak ($h_{m}$), and horizontal distance (dis) between an antenna and the peak.

It has been previously demonstrated that a significant improvement with the ADEM model can be achieved by using $E_{C}$ based on $E_{A}$, wherein a specific elevation range below $E_{M}$ is rejected. However, the rejection range $E_{C}$ should not be a constant value since there are different heights of vegetation and changeable distances between antennas and objects. To estimate the relationships among the range, the height of a mountain peak, and the horizontal distance between antennas and the peak, we assumed that the antenna was mounted beside the peak of a mountain, as shown in Figure 3.

As shown in Figure 3, the term $e_{w}$ can be used to denote the realistic range of observations that should be rejected because of multipath errors, and dis denotes the horizontal distance between the antenna and peak; the terms $h_{t}$ and $h_{m}$ denote the height of dispersed obstacles (i.e., trees) and the height of the mountain peak, respectively. According to their geometric relationships, the term $e_{w}$ can be expressed as follows:

(4) $$e_{w}=\arctan\left(\frac{dis\cdot h_{t}}{dis^{2}+h_{t}h_{m}+h_{m}^{2}}\right).$$

**Figure 4:**
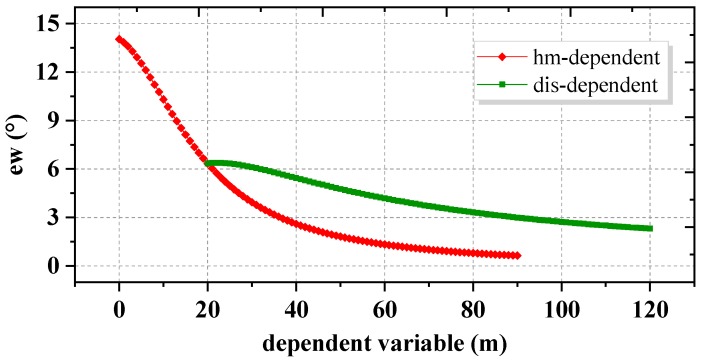
The relationships among $e_{w}$, $h_{m}$, and dis.

In the ADEM model, the rejected range $e_{w}$ is instead by the constant value $E_{C}$. In fact, however, $e_{w}$ should be a variable because of the growth of vegetation throughout the year. For quantitative research, as shown in Figure 3, we approximately set the heights of trees $h_{t}$ as a constant value of 5 m, set dis as 20 m, and set $h_{m}$ as 20 m. Then, the geometric relations among $e_{w}$, $h_{m}$, and dis were visualized in Figure 4.

In Figure 4, the relationship between $e_{w}$ and $h_{m}$ is depicted by the red line, and the relationship between $e_{w}$ and dis is depicted by the green line. It can be seen in Figure 4 that the term $e_{w}$ decreases with the increasing height of the ridges $h_{m},$ and significant decreases can be found along the section (0–40 m). Results show that an exclusion may lead by the constant value $E_{C}$ in ADEM model. Here, a careful weighting method that employs the ADEW model is proposed to derive a virtual variable like $e_{w}$ so that bad observations near the mountain ridge can be rejected with high accuracy. Hence, the variance of observations, $\sigma^{2}$, can be modified as follows:

(5) $$\sigma^{2}=a^{2}+\frac{b^{2}}{\sin^{2}\left(E^{\prime }\right)},E^{\prime }=\left(\frac{E-E_{A}}{\frac{\pi }{2}-E_{A}}\right)\cdot \frac{\pi }{2}.$$

In Equation (5), the projection transformation is adopted to project the measured elevation (E) used in the stochastic model to a virtual elevation ($E^{\prime }$), that equaling to change the coefficients in the weighting model.

From Equation (5), an elevation angle in the range of [$E_{A}$, π/2] can be transformed to [0, π/2]. Note that in the ADEW model, the measured elevation angle E, which is lower than $E_{M}$ in the ADEM model, will instead be determined by the virtual elevation angle $E^{\prime }$, and for the results, the rejection range is changeable according to variations of $h_{m}$ as well as dis. Higher $h_{m}$ or longer dis values will lead to a narrower range of rejection with careful weighting. This improvement not only increases the positioning accuracy through the assignment of phase observations with careful weights, but also promotes the usage of available observations.

### 2.4. Processing Flowchart

**Figure 5:**
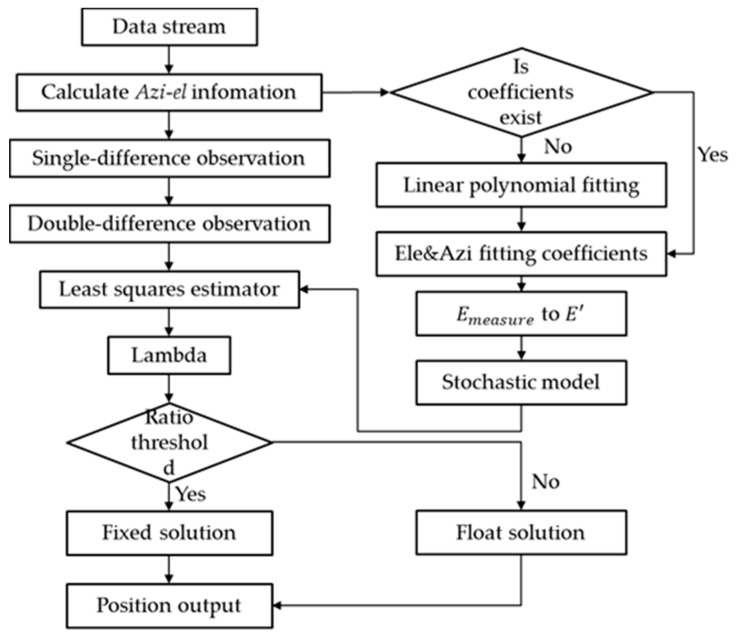
Flowchart of the real-time kinematic (RTK) technique under the ADEW model.

In order to realize micro-deformation monitoring in real-time, we developed a RTK platform, which can deal with heavy data streams and estimate coordinate biases in real-time. The main processing procedure is shown in Figure 5:

The procedure and ADEW model can be divided into three steps:

1. Over a session time of 24 h, GNSS observations were collected to compute all azimuth-elevations (*Azi-el*) for single positions given the approximate coordinates of the stations so that the ADEM coefficients could be initialized by $E_{min}$ for the first use.
2. Single-difference observations (SD) derived from raw observation data were applied to establish the DD observations. The float ambiguity of the DD ambiguity was estimated by the least-squares (LS) criterion, and the virtual elevation $E^{\prime }$ was used for careful weighting. To achieve a higher precision of the DD ambiguity, Lambda was employed to estimate the group of optimum integer ambiguities according to a threshold of the least-squares ratio (3.0).
3. Fixed coordinate biases were calculated as well as output by the optimum integer ambiguities if the solution was successfully fixed; otherwise, the float solution was used instead.

## 3. Experiments and Results

To assess the proposed method in a complex environment, a series of experiments were performed in two regions with frequent landslide occurrences. The data were processed by a GNSS software package developed by our research group, which can achieve millimeter-scale precision for deformation monitoring of a short-baseline within 10 km under an unsheltered environment with GNSS data. For comparison purposes, the SF model was also employed to evaluate our proposed method. Here, we present our results.

### 3.1. Data and Study System

**Figure 6:**
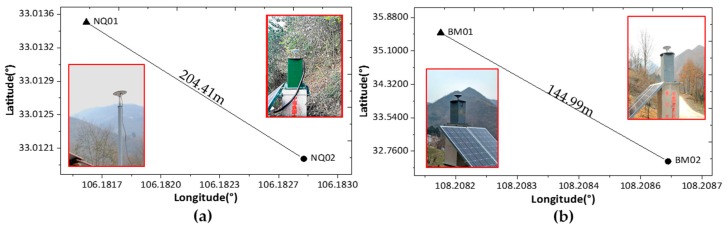
The distribution of stations for both sites: (**a**) reference station (NQ01) and rover station (NQ02); (**b**) reference station (BM01) and rover station (BM02).

Two sites in Shaanxi, China, were used as study areas to assess the proposed technique’s ability to improve the accuracy of micro-deformation monitoring results. One site was located in the city of NingQiang, while the other site was located in the city of HanZhong. Both surrounding environments are complex with mountainous terrain and various obstacles, which act as major sources of diffraction and multipath errors. At site (a), the GNSS observations were collected randomly on the days of year (DOY) 021, 045, and 072 (21 January, 14 February, and 12 March, respectively) in 2018. At site (b), the GNSS observations were collected on DOY 040 in 2018. The distributions of stations at sites (a) and (b) are shown in Figure 6.

As shown in Figure 6, the lengths of the baselines were 204.41 m and 144.99 m for sites (a) and (b), respectively. The rover station (NQ02) at site (a) was installed beside a hillside that was severely impacted by ridges and trees, especially from 0 to 180° in the azimuth direction (shown in Figure 1). The rover station (BM02) at site (b) was installed in an area with mountainous ridges nearby. The reference stations at both sites were mounted in better places, and the equipment used was the same as that used at the rover sites. The operational details for the receivers and antennas used in the test, which are capable of tracking BDS (B1, B2, B3), GPS (L1, L2, L5), and GLONASS (P1, P2) satellite signals, are summarized in Table 1.

The following three different model approaches were employed: (1) a conservative cut-off elevation angle was used with the TFC model; (2) a fixed cut-off elevation based on the ADEM was used; and (3) the proposed ADEW model was used. In addition, the SF model, which realizes the results in the coordinate domain in a simple manner, was employed to allow for comparisons with the ADEM model results. In our experiments, dual-frequency observations from GNSS were processed by our program in a simulated real-time mode, which means that the data stream was input to the platform epoch by epoch; the results and output were also estimated epoch by epoch.

The data collected from site (a) are discussed in Section 3.2, Section 3.3, Section 3.4 and Section 3.5, and the data collected from site (b) are discussed in Section 3.6 of this manuscript. As was mentioned earlier, there was no significant motion during the selected session time, and thus, the coordinate bias time series were caused solely by noise and propagation effects, such as those due to diffraction and multipath errors.

### 3.2. $E_{AziEle\;mask}$ Modeling and Performance Analysis

**Figure 7:**
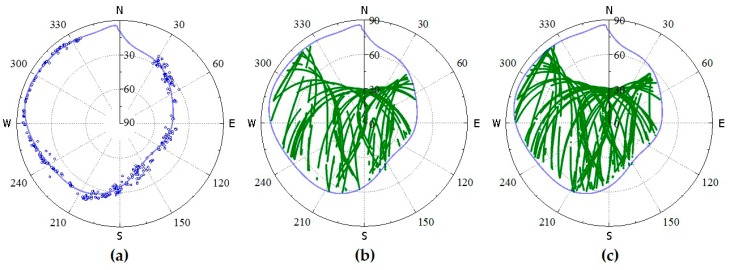
ADEM model fitted by the polynomial method and the compared sky view of satellites: (**a**) Initial and terminal point and fitted line; (**b**,**c**) the sky view of satellites at DOY 045 and 072, respectively.

Figure 7 shows the curve of the line fitted with the $E_{min}$ points of all satellite traces received by rover station NQ02 on DOY 021. The other data from DOY 045 and 072 were used to assess the reliability of the curve line. As Figure 7a clearly shows, the scatter points were evenly distributed on both sides of the fitted-line curve.

From Figure 7, it can be observed that the fitted line coincides with the boundary of the satellite tracking on the selected days, which indicates that the specific fitted coefficients are suitable for a long period of time without considering the sidereal cycle. In this study, the coefficients were renewed at 00:00 UTC on the first day of each month.

### 3.3. Performance Evaluation of ADEM

**Figure 8:**
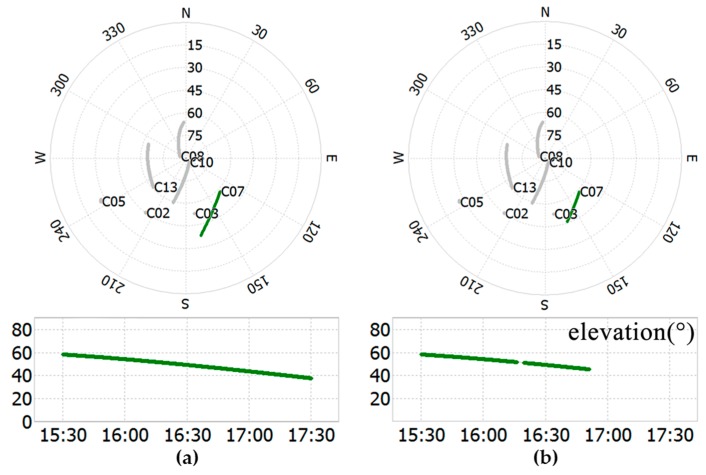
Sky view of points from 15:30 to 17:30 and the satellite elevation of C07: (**a**) reference point NQ01 and (**b**) rover point NQ02.

Geostationary earth orbit (GEO) and inclined geosynchronous orbit (IGSO) were used, which could be tracked by the antenna at all times. Therefore, only the observations of BDS are considered in this section, as well as in Section 3.4. The observation session time ranged from 15:30 UTC to 17:30 UTC on DOY 021 in 2018. The sky views of common satellites received both by NQ01 (a) and NQ02 (b) are shown at the top of Figure 8a,b, and the elevations of satellite C07 are also given at the bottom of Figure 8a,b.

**Figure 9:**
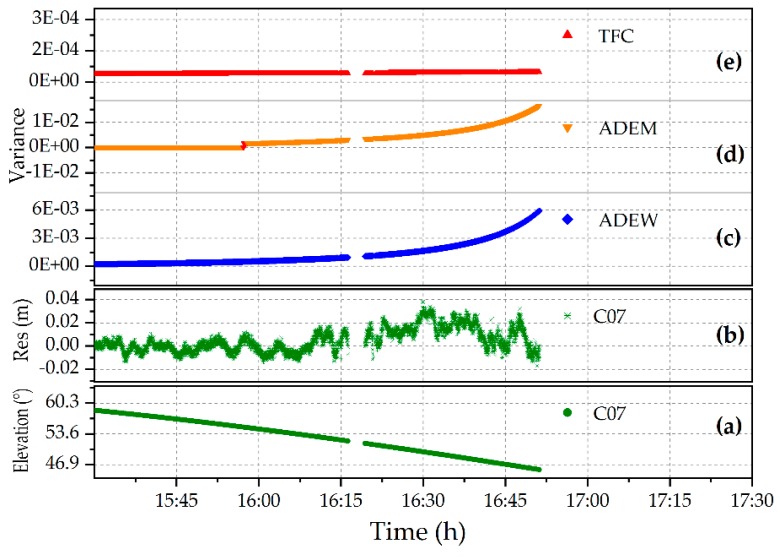
DD residuals and the elevation time series of C07, as well as the corresponding variance in the different models: (**a**) elevation angle; (**b**) DD residuals; and variance in (**c**) ADEW; (**d**) ADEM; and (**e**) TFC.

It is clear from Figure 8 that the signal of NQ02 was lost around 16:50, but the signal was still traced by NQ01. According to the panoramic view of NQ02, the signals of C07 were affected by the vegetation at about 16:10, and the influence lasted until the signal was lost in the set behind the mountain peak at around 16:50, which is where the azimuth-elevation was (168.25°, 45.51°). In the experiments, the satellite C03, which has a higher elevation angle (about 53°) and is not readily influenced by the multipath, was taken as the reference satellite to avoid a change of the reference satellite. The DD residuals and elevation angle of C07 from 15:30 to 16:50 and the variances of the TFC, ADEM, and ADEW models in stochastic models are shown in Figure 9.

**Figure 10:**
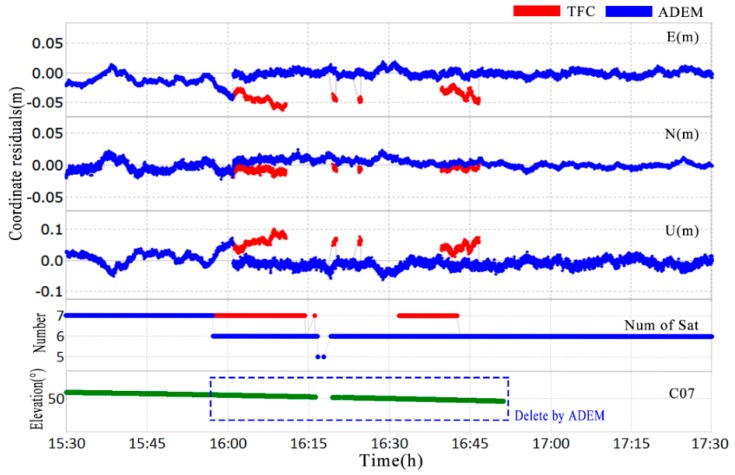
Deviation of the fixed coordinate time series using the TFC method (red) and the ADEM method (blue) from 15:30 to 17:30 for NQ02 (**top**); the number of satellites used for the estimation (**middle**); the session dealing with C07 for the ADEM method (**bottom**).

It is clear from Figure 9 that the residuals significantly deviated from zero between 16:10 and 16:50 with multipath errors, which is when the elevation angles of the satellites were near the mountain ridge. This could have led to a lower accuracy of positioning, or even failures in the integer ambiguity fixing. Therefore, it was necessary to weaken the effects with a reasonable weighting strategy implemented by the variance. Noting that the variance time series of the ADEM model climbed steeply, while the variance of the TFC model changed slowly with the elevation reduction of C07. With a conservative cut-off elevation of 15°, the time series of the coordinate bias in real-time was compiled, and the results are shown at the top of Figure 10; the elevation angle and the numbers of valid satellites are also shown in this figure.

A major discrepancy can clearly be seen between 15:55 and 16:50, whereby the deviation timeseries of the TFC model (red) exceeded that of the ADEM model (blue), and even the integer ambiguity could not be fixed by the TFC during 16:15 to 16:30 as a result of the impact of multipath errors. With the reduction of bad observations near the mountain ridge achieved by using the ADEM model, the root mean square (RMS) of the coordinate bias in the horizontal direction of the time series improved by 18.91% (from 0.1396 to 0.1132 m), and the RMS in the vertical direction improved by 34.93% (from 0.0272 to 0.0177 m). The success rate for integer ambiguity fixing improved from 63.4 to 87.8%.

**Figure 11:**
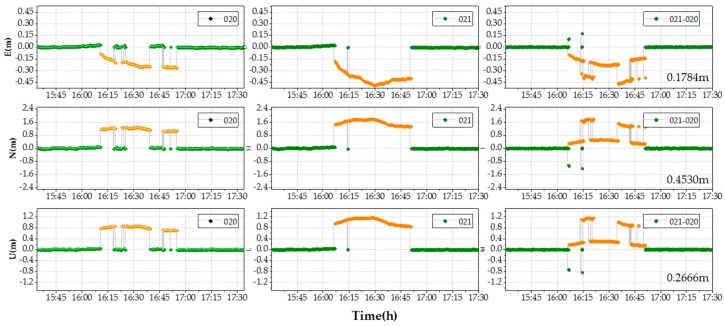
Coordinate bias in ENU (east, north, up) direction: E (top line), N (middle line), and U (up line). The RMS of the coordinate bias in the top right corners at right column: DOY 020 (left column), DOY 021 (middle column), and after correction of the SF model (right column).

Next, we compared the ADEM model results with those from the SF approach to evaluate the performance in terms of positioning accuracy in the coordinate domain for deformation monitoring within the study environments. In this test, we adopted the first day (DOY 020) to build the SF model. The db8 wavelet was used to filter the high frequency noise [41,42], and filter value subtraction was performed at the sidereal (23 h 56 min 04 s) [40]. The data at DOY 021 were set as the multipath reduction target. Experimental results are shown in Figure 11.

Figure 11 shows the coordinates bias in each ENU direction before and after the multipath correction at DOY 021 for the SF model, both with ambiguity fixed successfully (green) and failed (orange) solutions. The RMS of the correction coordinate bias reached up to 0.1784 m, 0.4530 m, and 0.2666 m in the E, N, and U direction, respectively. It is clear that the performance of the SF model as shown was not very good according to the data from our experiment, since the ambiguities in the repeat period were not fixed every time. In fact, the integer ambiguities with the data collected from high-multipath environments were difficult to fix. Therefore, the SF model may not be suitable for use in such complex environments, and the following work only shows the typical model results for comparative purposes.

### 3.4. Performance Evaluation of the ADEW Model

**Figure 12:**
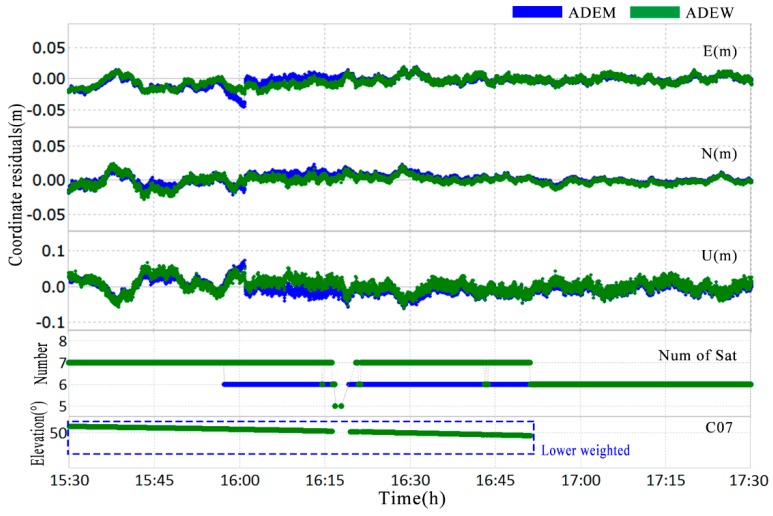
Deviation of the fixed coordinate time series using the ADEM method (blue) and the ADEW method (green) from 15:30 to 17:30 of NQ02 (**top**); the number of satellites used for the estimation (**middle**); the session dealing with C07 in the ADEW method (**bottom**).

Although the ADEM model (blue) performed better than the TFC (red), as shown in Figure 10, there was still a break point detected at around 15:55 UTC. The break may be accounted for by the discontinuous variance of C07 (Figure 9d) used in the stochastic model. To overcome the limitations discussed in Section 3.3, the ADEW model is proposed, and it uses projection transformation for a continuous variance without considering the heights of mountains and the distances between the obstacles and the antennas. The variance is shown in Figure 9c, and the improvement in terms of positioning is presented in Figure 12.

Figure 12 shows that the RMS values for the ADEM model were 0.0096 m and 0.0272 m in the horizontal and vertical direction, respectively. In comparison, the deviation timeseries of the ADEW model performed better, with RMS values of 0.0075 m and 0.0191 m in the horizontal and vertical direction, respectively. Not only did the RMS improve by 21.9% and 29.8% in the horizontal and vertical direction, respectively, but the usage of observation data improved by 6.25% as well. These findings indicate that the performance of the ADEW model is better than that of the ADEM model.

### 3.5. Performance of the ADEW Model with GNSS Data

**Figure 13:**
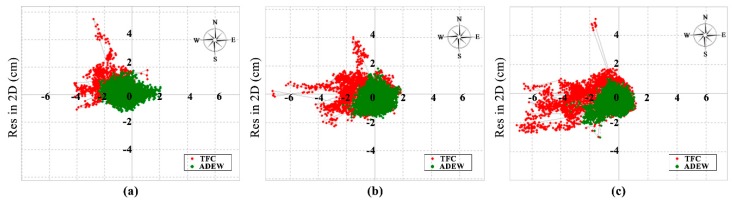
Deviation of the fixed coordinate time series in the horizontal direction with GNSS observations from NQ02 for three different days: (**a**) DOY 021; (**b**) DOY 045; (**c**) DOY 072.

The experiments presented above were assessed with BDS observations. Next, three groups of GNSS observations collected on random days for NQ02 were employed to further assess the performance of the proposed ADEW model. The navigation systems used in these experiments consisted of GPS, GLONASS, and BDS. Figure 13 illustrates the deviation of the coordinate bias in the time series for the horizontal direction derived from a fixed solution on DOY 021 (a); 045 (b); and 072 (c) in 2018.

The results shown in Figure 13 indicate that the ADEW model (green) exhibited a lower RMS than the TFC model (red); details of the RMS values are shown in Table 2.

It is clear that the RMS of the coordinate bias with the ADEW model was found to have improved, relative to that of the ADEM model, by 30.11%, 37.27% and 37.71% in the horizontal direction on DOY 021, 045, and 072, respectively. The mean improvement of the RMS in the vertical direction was greater than 31.58%. Furthermore, the results suggest that the polynomial coefficients of the ADEM model are applicable for a long period of time, which can help to mitigate the effects of diffraction and multipath errors, even within a full month.

### 3.6. Performance of ADEW with another Rover Point

**Figure 14:**
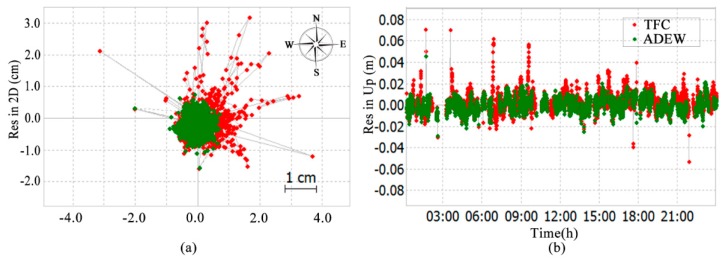
Deviation of the fixed coordinate time series in the horizontal (**a**) and vertical (**b**) direction via GNSS observations from BM02 on DOY 040.

To validate the ability of the ADEW model in another complex environment, the rover point (BM02) at site (b) was analyzed. The observations of GNSS were collected on DOY 040 in 2018, and the polynomial coefficients were estimated by using the polynomial fitted model. Details of the receivers and antennas used have been given in Table 1. Figure 14 shows the time series of the deviation of coordinate bias in the ADEW model (green), and the results for the TFC model (red) are also shown in Figure 14 for comparison.

As shown in Figure 14, the RMS values of the coordinate bias time series obtained by using the ADEW model were 0.0095 m and 0.0069 m in the horizontal and vertical direction, respectively. Use of the TFC model resulted in RMS values of coordinate bias of 0.0116 m and 0.0076 m in the horizontal and vertical direction, respectively. Thus, significant improvements of 18.10% and 9.21% were achieved by applying the ADEW model over the ADEM model.

## 4. Discussion

As the area surrounding a GNSS antenna remains unchanged for a relatively long period, this study constructed a physical elevation mask model around antennas by using multi-navigation satellites systems. Model experiments were then used to demonstrate the performance of this approach for high-precision micro-deformation monitoring in complex environments, such as those where landslides occur.

The specific coefficients of the ADEM model presented in this study were estimated by a 24 h session of GNSS observations collected from NQ02 in the complex environment of site (a). Results of these experiments indicated that the specific coefficients were applicable for DOY 021, 045, and 072 in 2018. Subsequently, the performance of the ADEM model was investigated by using BDS observations, and we compared the results to those obtained with the SF model. Results of these experiments revealed that the RMS values of coordinate bias when using the ADEM model had improved by 18.91% and 34.93% in the horizontal and vertical direction, respectively, in comparison with those derived from the use of the TFC model; significant improvements over the SF model were also observed. However, a break point remained, and this could be accounted for by the rejection of observations caused by the fixed cut-off elevation.

In order to overcome the limitations of the TFC and ADEM models, the range of impacts caused by the cut-off elevation related to $h_{m}$ and *dis* were investigated. Detailed analyses revealed that the adaptive range was greatly affected by $h_{m}$ but not by dis. Therefore, the ADEW model, in which the projection transformation is employed to project the satellites elevation range from [$E_{AziEle\;mask}$, π/2] to a virtual range [0, π/2], was developed by careful weighting in a stochastic model. The model experiments with BDS observations revealed that the RMS values of the ADEW model had further improved by 21.9% and 29.8% in the horizontal and vertical direction, respectively. Model experiments with GNSS observations demonstrated mean RMS improvements of 34.69% and 31.58% in the horizontal and vertical direction, respectively. It was also demonstrated that the physical coefficients of the ADEW model are applicable for a long period of time, even more than a month. Finally, another rover station BM02 at site (b) was used to assess the validity of the ADEW model. The results of this analysis agreed in that the ADEW model can significantly reduce the effects of diffraction and multipath errors by 18.10% and 9.21% in the horizontal and vertical direction, respectively.

In summary, the proposed algorithm has been demonstrated to not only improve the positional accuracy, but also the usage of available observations, and it can achieve accuracies ranging from the centimeter-scale to the millimeter-scale in complex environments. Additionally, the coefficients of ADEM used in the ADEW model have been demonstrated to be available for a long session without the need to consider the sidereal cycle.

## 5. Conclusions

GNSS techniques are recognized as an effective way to monitor geologic deformation in real-time. However, the signals of satellites are severely affected by both concrete and dispersed obstacles in complex environments; thus, the results of many existing models such as the SF model can lead to failures in interpretations. This study presents an ADEW model based on the specific ADEM surrounding antennas. The experiments on BDS observations demonstrated that the RMS of coordinate bias using the ADEM model improved relative to that of the TFC model by 18.91% and 34.93% in the horizontal and vertical direction, respectively. The extended ADEW model conferred a further improvement of 21.9% and 29.8% in the horizontal and vertical direction, respectively, when compared to the results from the ADEM model. The following conclusions can be drawn from the experimental results and validation work:

1. Multi-GNSS methods provide a convenient way to model a physical elevation mask for landslide monitoring in complex environments, and such an approach improves upon the traditional method that relies on theodolite measurements.
2. The ADEM model constructed by GNSS data instead of geoid data can greatly reduce the impact of physical obstacles near the antenna in complex environments.
3. The ADEW model can be used to further improve the performance of micro-deformation monitoring in complex environments relative to the ADEM model as it has been demonstrated here to exhibit not only lower RMS values of coordinate bias in the time series, but also increases the usage of available observation data.

The ADEW model depends upon specific coefficients for each ADEM. Therefore, developing a universal algorithm to eliminate the effects in real-time will be the goal of our future work. We will also consider the dilution of precision (DOP) of GNSS satellites in the future.

## Figures and Tables

**Table 1:** Monitoring datasets used for the investigation, and the types of receivers and antennas used. (REF and ROV denote the reference station and rover station, respectively).

Site	(a)		(b)	
Station	REF(NQ01)	ROV(NQ02)	REF(BM01)	ROV(BM02)
**Baseline length [m]**	204.41		144.99	
**Receiver**	UNICORECOMM-UR380 (GPS + GLO + BDS)			
**Antenna**	MEASURING ANTENNA (GPS + GLO + BDS)			
**Sampling rate [s]**	1			
**Elevation cut-off [°]**	0			
**Ratio threshold**	3			

**Table 2:** Standard deviation (RMS) and improvement value in the horizontal direction (2D) and vertical (U) coordinate error of the 3 different days derived from the TFC and ADEW models.

DOY	RMS-2D (m)			RMS-U (m)		
	TFC	ADEW	Improved	TFC	ADEW	Improved
021	0.0109	0.0055	30.11%	0.0250	0.0168	32.80%
045	0.0108	0.0068	37.27%	0.0211	0.0124	41.23%
072	0.0100	0.0062	37.71%	0.0228	0.0158	30.70%
